# The Effect of Proline *cis*-*trans* Isomerization on the Folding of the C-Terminal SH2 Domain from p85

**DOI:** 10.3390/ijms21010125

**Published:** 2019-12-23

**Authors:** Francesca Troilo, Francesca Malagrinò, Lorenzo Visconti, Angelo Toto, Stefano Gianni

**Affiliations:** 1 Istituto Pasteur—Fondazione Cenci Bolognetti, Dipartimento di Scienze Biochimiche “A. Rossi Fanelli”, 00161 Roma, Italy; francesca.troilo@uniroma1.it (F.T.); francesca.malagrino@uniroma1.it (F.M.); lorenzo.visconti@uniroma1.it (L.V.); angelo.toto@uniroma1.it (A.T.); 2 Istituto di Biologia e Patologia Molecolari del CNR, Sapienza Università di Roma, 00185 Rome, Italy

**Keywords:** protein folding, misfolding, kinetics, mutagenesis

## Abstract

SH2 domains are protein domains that modulate protein–protein interactions through a specific interaction with sequences containing phosphorylated tyrosines. In this work, we analyze the folding pathway of the C-terminal SH2 domain of the p85 regulatory subunit of the protein PI3K, which presents a proline residue in a cis configuration in the loop between the βE and βF strands. By employing single and double jump folding and unfolding experiments, we demonstrate the presence of an on-pathway intermediate that transiently accumulates during (un)folding. By comparing the kinetics of folding of the wild-type protein to that of a site-directed variant of C-SH2 in which the proline was replaced with an alanine, we demonstrate that this intermediate is dictated by the peptidyl prolyl cis-trans isomerization. The results are discussed in the light of previous work on the effect of peptidyl prolyl cis-trans isomerization on folding events.

## 1. Introduction

A detailed description of the mechanism by which a polypeptide chain achieves its functionally active conformation is one of the central problems of protein science [1,2,3,4,5,6]. In fact, it is well-established that aberrant folding into non-native misfolded conformations may lead to potentially devastating events that lead to severe human pathologies [7,8,9]. Our current understanding of protein folding is predominantly based on the study of small single-domain proteins, with a specific focus on elucidating the general rules of folding, as well as on identifying the recurring features of misfolded traps [10,11,12,13,14,15,16,17].

In general, protein misfolding is initiated by the transient accumulation of non-native contacts. These interactions may either spontaneously break, with the protein folding towards its native conformation, or drag the polypeptide chain towards long-lived misfolded conformations that may potentially lead to aggregation [7,8,9]. Because of the links between aggregation and disease, the study of misfolding phenomena is of particular importance and much experimental effort has been devoted to understanding them.

One of the most well-known examples of transient misfolding is caused by the cis-trans isomerization step of the peptide bond involving proline residues [18,19,20,21,22]. In fact, whilst prolines are generally found in the trans conformation in the native state of proteins, under denaturing conditions, the trans configuration of X-Pro peptide bonds is usually only slightly favoured over the cis configuration [23]. The denatured state may therefore populate both the cis- and trans-conformations, depending on their specific amino acidic sequence. Since the cis-trans isomerization steps of the X-Pro peptide bond are often slower than protein folding, the presence of an incorrect isomerization of Pro residue in the denatured state may lead to a kinetic complexity that is characterized by multiple kinetic phases and transient misfolding [21].

**Figure 1:**
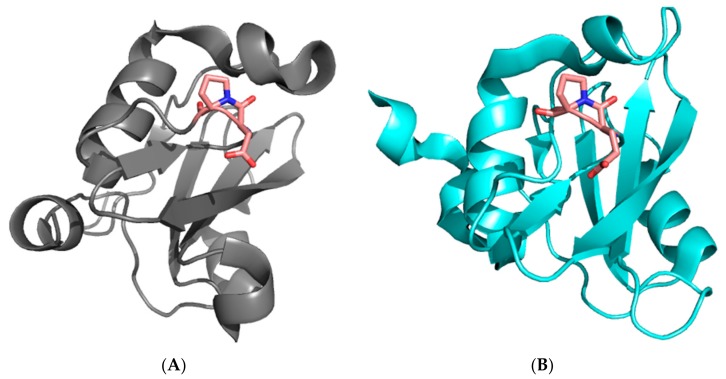
Three-dimensional structure of the N-SH2 (**A**) and C-SH2 (**B**) domains of the subunit p85 of PI3K. The residues D73–P74 of the N-SH2 domain and E683–P684 of the C-SH2 with the peptide bond in a cis conformation, located in a loop between the βE and βF strands, are highlighted in sticks.

SH2 domains are conserved domains of about 100 amino acids, whose biological role lies in recognizing and binding specific sequences that contain phosphorylated tyrosine residues [24,25,26]. The protein p85 regulates the catalytic activity of the protein PI3K and mediates its interactions with various receptor tyrosine kinases through physical recognition between its two SH2 domains (the N-SH2 and C-SH2 domains, separated by a coiled coil region) and a consensus pYxxM sequence. Both the N-SH2 and C-SH2 domains present a characteristic X-Pro peptide bond in a cis configuration in a loop between the βE and βF strands (Pro74 in the case of N-SH2 and Pro684 in the case of C-SH2, see Figure 1). We recently analyzed the folding mechanism of N-SH2 and demonstrated that the presence of such a cis X-Pro peptide bond has a remarkable effect on the folding of the protein [27]. In fact, we observed an apparent mismatch between folding and unfolding rate constants. This behavior, which to the best of our knowledge has not been reported for the folding of a single-domain protein, could be easily interpreted by invoking a structural heterogeneity in the denatured state.

Here, we present the kinetic folding mechanism of C-SH2. By employing single and double jump folding and unfolding experiments, we demonstrate the presence of an on-pathway intermediate that transiently accumulates during (un)folding. The intermediate, which is due to a heterogeneous structural conformation of the protein, is revealed from the presence of double exponential folding and unfolding time-courses. Surprisingly, we show that mutating Pro684 to alanine abolishes the accumulation of the intermediate, restoring single exponential (un)folding. This finding suggests that the Glu–Pro peptide bond in a cis configuration determines the accumulation of the on-pathway intermediate, an observation that contrasts with what was previously observed in the homologue’s N-SH2 domain, as well as what is typically observed in single-domain proteins.

## 2. Results and Discussion

**Figure 2:**
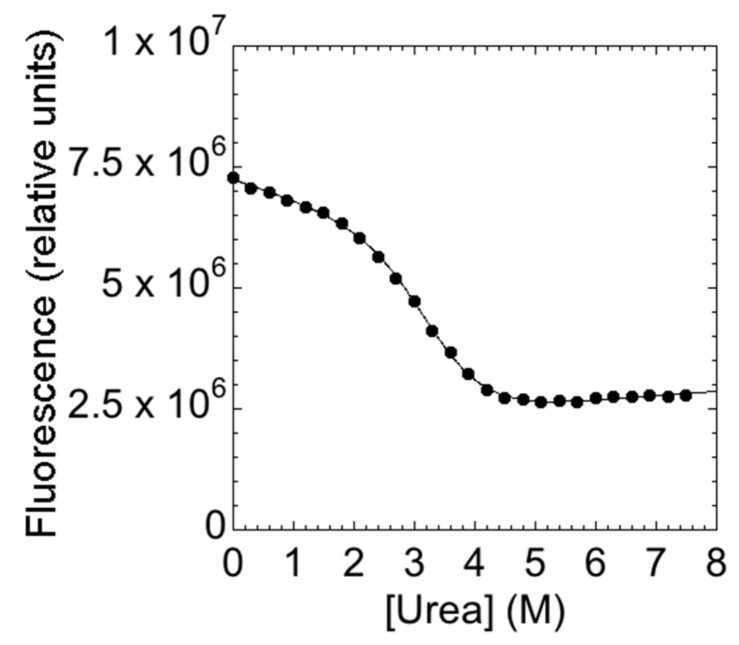
The equilibrium denaturation experiment of C-SH2 performed in 50 mM Hepes buffer at pH 7.4 and 25 °C. The denaturant agent used was urea. The change in the intrinsic fluorescence of the tryptophan residue versus urea concentrations is consistent to a two-state transition.

The main aim of this study is to provide a description of the folding properties of C-SH2. Consequently, we conducted urea-induced folding and unfolding experiments, both at equilibrium and time-resolved. The urea-induced unfolding of the C-SH2 monitored at equilibrium at pH 7.4 is reported in Figure 2.

It is evident that the observed transition conforms to a simple two-state profile, which would suggest that no stable intermediate accumulated at equilibrium [14]. A quantitative analysis of the (un)folding profile returns an *m*_D-N_ value of 1.9 ± 0.1 kcal mol^−1^·M^−1^, which is consistent with a protein of 108 amino acids [28], and an apparent midpoint of 2.8 ± 0.1 M. A global analysis of the fluorescence spectra, obtained at different urea concentrations, further confirmed the robustness of the apparent two-state behavior. Similar folding parameters were obtained when calculated using data recorded at different wavelengths.

**Figure 3:**
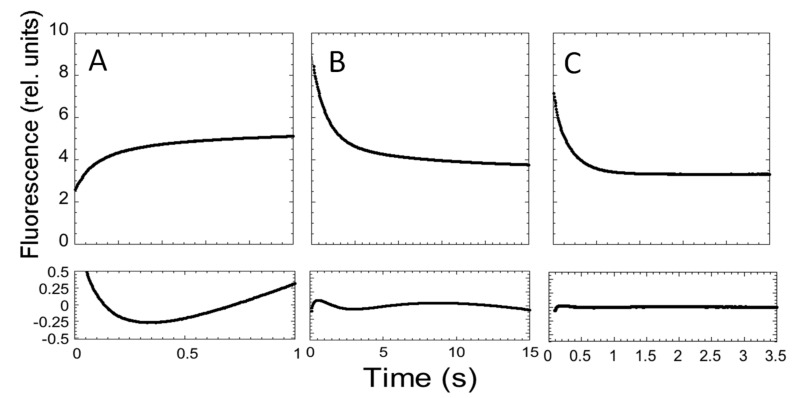
Folding and unfolding kinetics of C-SH2. (**A**) Refolding time course of C-SH2 measured in 50 mM Hepes buffer at pH 7.4 in the presence of 0.37 M urea. (**B**) Unfolding time course of C-SH2 measured in the presence of 3 M urea. (**C**) Unfolding time course of C-SH2 measured in the presence of 7.27 M urea. Below each panel we report the residuals to a single exponential decay.

In an effort to describe the kinetic properties of the folding of C-SH2, we carried out fluorescence-monitored stopped-flow mixing experiments at pH 7.4 and 37 °C. At all of the monitored urea concentrations, the refolding time courses were consistent with a double exponential decay. Conversely, in the case of unfolding, whilst two distinct kinetic phases could be observed up to a urea concentration of 4 M, when the native protein was mixed with solutions containing a higher urea concentration, only a single exponential could be observed (Figure 3).

**Figure 4:**
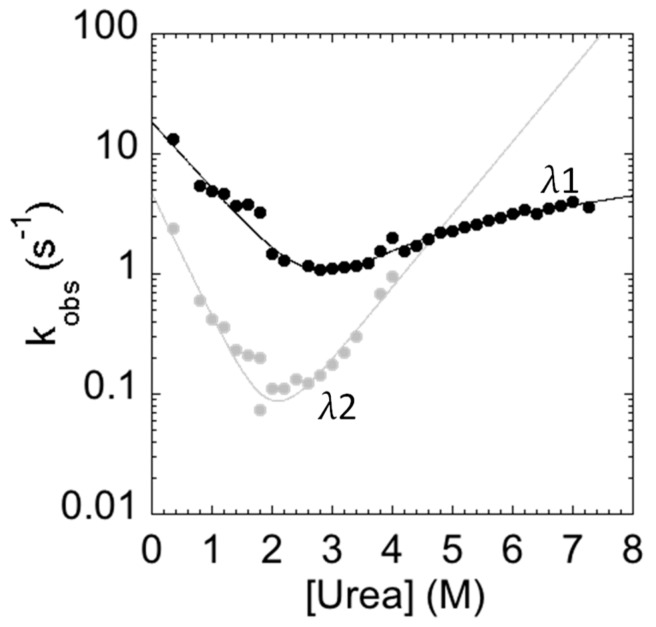
Folding kinetics of the C-SH2 domain measured in 50 mM Hepes buffer at pH 7.4 and 37 °C. λ1 represents the fast phase of the process (in black); λ2 represents the slow phase of the process (in gray). The unfolding arm shows a monophasic behavior from 8 to 4 M urea and a biphasic behavior up to 4 M urea. The refolding arm shows a biphasic behavior.

The measured folding and unfolding rate constants for C-SH2 are reported in Figure 4. It is of interest to analyze the dependence of the unfolding profiles. In fact, inspection of the urea dependence of the two kinetic phases λ1 and λ2 indicates that, when the value of λ2 exceeds that of λ1, the apparent unfolding conforms to a single exponential. This observation suggests that λ1 and λ2 are two sequential steps and demands additional consideration.

In general, the presence of multiphasic kinetics may either arise from the accumulation of a transient intermediate or from a structural heterogeneity of the ground state(s) [29,30,31,32,33,34,35,36,37,38]. In the case of C-SH2, it is interesting to observe that a clear biphasic behavior can be observed up to 4 M (urea), both in the refolding and in the unfolding arms. A quantitative analysis of the observed dependence of λ2 returns an apparent midpoint of this phase at a urea concentration of 2.5 M. Therefore, accumulation of the intermediate in unfolding experiments at urea concentrations higher than 3 M should be negligible and unfolding should thus conform to a single exponential. On the basis of these observations, it would appear that multiphasic unfolding should arise, at least in part, from a structural heterogeneity of the native state.

**Figure 5:**
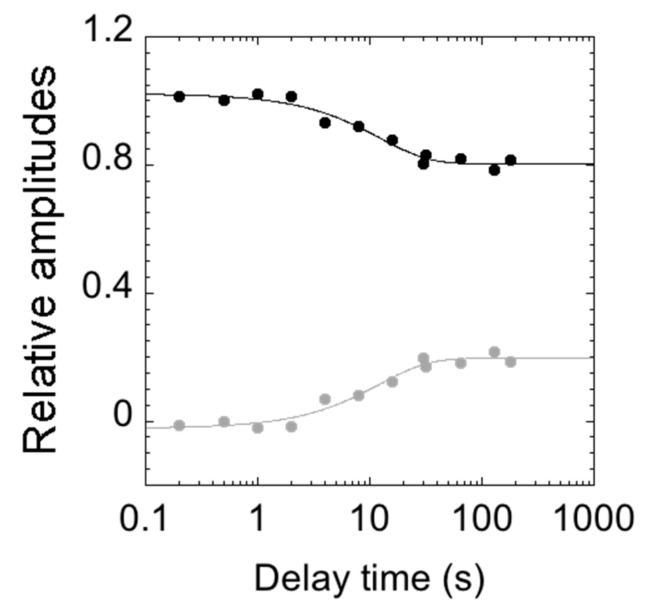
Relative amplitudes of the observed rate constants calculated from double mixing refolding experiments at different delay times between the first and second mix (in logarithmic scale). The relative amplitudes of the slow unfolding phase are represented in gray, and those of the fast phase in black. At short delay times, unfolding consists of a single exponential behaviour (with amplitudes approaching 0). Upon increasing the delay time between the first and second mix, the unfolding becomes consistent with a double exponential behaviour (the amplitudes of the slow phase increase).

To infer the structural plasticity in the native state of C-SH2, we resorted to performing double jump interrupted refolding experiments. In particular, the denatured protein at 4 M urea was first mixed in a 1:1 dilution experiment with refolding buffer; then, after a controlled delay time, refolding was interrupted by challenging the protein with an unfolding solution, leading to the final concentration of 3 M [urea]. All of the measured time courses were globally fitted to a double exponential decay, with shared rate constants. Figure 5 reports the relative unfolding amplitudes of the observed time courses as a function of the delay time between the first and the second mix.

Interestingly, at short delay times, unfolding conformed to a single exponential behavior, with the slow unfolding phase having a negligible amplitude. Increasing the delay times resulted in an increase in the amplitude of the slow unfolding phase with an apparent rate constant of circa 0.1 s^−1^, which is consistent with the slow refolding phase observed in single-mix refolding experiments. Following these observations, a plausible scenario to account for the observed data may be that the slow unfolding phase is associated with the accumulation of a slow refolding species. Under such conditions, the complex kinetics reflect a heterogeneity in the native state of C-SH2. Remarkably, however, since it is observed that, when the value of λ2 exceeds that of λ1, the apparent unfolding conforms to single exponential (Figure 4), it may be concluded that the fast and slow unfolding species are on the same reaction path and, therefore, report of two sequential steps. Therefore, the alternative conformation that can be found in the native state of C-SH2 acts as an on-pathway intermediate in its (un)folding profile.

A previous NMR analysis on C-SH2 also suggested that the protein has structural heterogeneity [39]. This structural variability was pinpointed by studying the exchange broadening of several side-chains of the domain, and alternative structural conformers were observed, particularly in the loop connecting the βE and βC strands, the βE and βF strands, and the βB and βG strands. By analyzing these loops, we noted that the sequence connecting the βE and βF strands contains a proline residue that was subsequently seen to populate a cis configuration in its peptide bond, when studied in the presence of a physiological ligand. Since the kinetics of the cis-trans isomerization step are typically slow and could be compatible with the rate constants observed in the slow kinetics of folding of C-SH2, we produced the P684A mutant to investigate the role of Pro684 in C-SH2 folding specifically.

**Figure 6:**
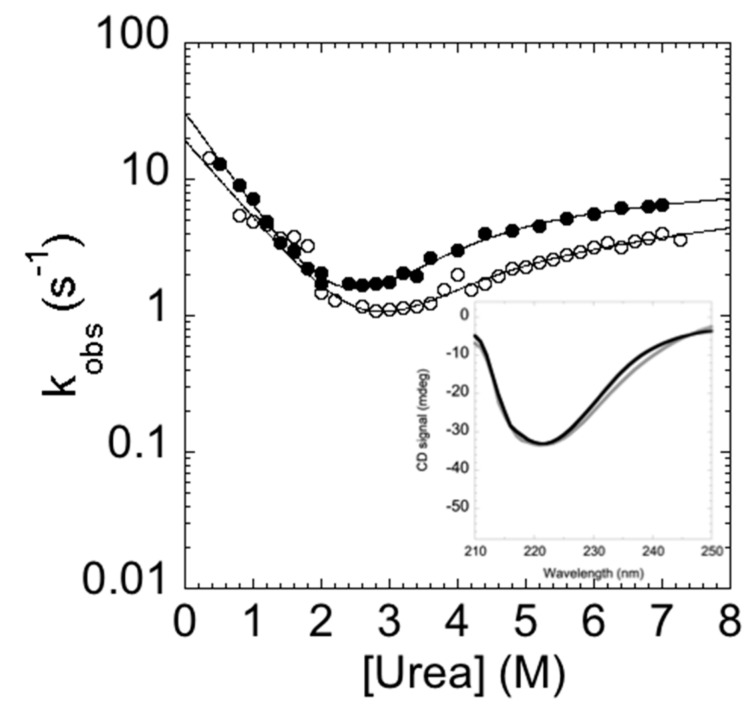
Chevron plot of the double mutant C-SH2 P684A (black circles) compared with the fast phase of the C-SH2 (empty circles). Upon the substitution of the proline with alanine, the slow phase is no longer present and the chevron plot resembles the fast phase observed for the C-SH2. Inset panel. Comparison of the far UV circular dichroism (CD) spectra of C-SH2 P684A (black) and C-SH2 (gray). It is evident that the mutation results in a marginal change in the secondary structure of the domain.

In analogy to the experiments performed on the C-SH2, the folding and unfolding kinetics of the P648A mutant were measured by stopped-flow at 37 °C in 50 mM Hepes buffer, pH 7.4. Remarkably, under all of the investigated experimental conditions, folding and unfolding appeared to be consistent with a single exponential behavior, indicating that the apparent complexity in the folding and unfolding kinetics of C-SH2 arises from a proline cis-trans isomerization event. To further support this view, it is interesting to observe that the chevron plot of P648A (Figure 6) closely resembles the fast (un)folding phase λ1 observed for C-SH2, whereas λ2 is absent.

The kinetic folding parameters that were calculated for C-SH2 and for the C-SH2 P684A variant are reported in Table 1.

## 3. Methods

### 3.1. Site-Directed Mutagenesis

The construct encoding the C-SH2 domain of the p85 subunit of the PI3K protein was subcloned in a pET28b+ plasmid vector. In order to avoid the dimerization of the domain due to the presence of a Cysteine in position 656, this residue was mutated in Serine. The construct encoding the C656S mutant was obtained using the gene encoding the C-SH2 wild-type (wt) as a template to perform a site-directed mutagenesis with the QuickChange Lightning Site-Directed Mutagenesis kit (Agilent technologies) according to the manufacturer’s instructions. The double mutant C656S-P684A was obtained through the same protocol using the pseudo-wt sequence C-SH2 C656S as a template. The mutations were confirmed by DNA sequencing.

### 3.2. Protein Expression and Purification

The expression of both C-SH2-C656S and C656S-P684A was performed in *Escherichia coli* BL21 cells. Bacterial cells were grown in LB medium, containing 30 μg/mL of kanamycin, at 37 °C until OD_600_ = 0.6−0.8, and then protein expression was induced with 1 mM IPTG. After induction, cells were grown at 37 °C overnight and then collected by centrifugation. To purify the protein, the pellet was resuspended in 50 mM TrisHCl buffer, 300 mM NaCl, and 10 mM Imidazole at pH 7.0 with the addition of an antiprotease tablet (Complete EDTA-free, Roche). After sonication and centrifugation, the soluble fraction from the bacterial lysate was loaded onto a nickel-charged HisTrap Chelating HP (GE Healthcare) column equilibrated with 50 mM TrisHCl, 300 mM NaCl at pH 7.0, and 10 mM Imidazole. The protein was then eluted by imidazole with a gradient from 0 to 1 M using an AKTA-prime system. Fractions containing the protein were collected and the buffer was exchanged to 50 mM Hepes at pH 7.4 and 300 mM NaCl by using a HiTrap Desalting column (GE Healthcare). The purity of the protein was analyzed through SDS-PAGE.

### 3.3. Equilibrium Unfolding Experiments

The equilibrium unfolding experiments on the pseudo wt C-SH2 C656S were carried out on a Fluoromax single photon counting spectrofluorometer (Jobin-Yvon, NJ, USA), by mixing the native protein (3 μM) with increasing urea concentrations. Experiments were performed at 25 °C, using a quartz cuvette with a path length of 1 cm, 50 mM Hepes buffer, and 300 mM NaCl at pH 7.4, and the intrinsic tryptophan emission was measured. The excitation wavelength was 280 nm, and fluorescence spectra were recorded between 300 and 400 nm. Data were fitted using Equation (1):

(1) $$Y_{obs}=\left(Y_{N}+Y_{D}\right)\frac{e^{mD-N\left(\left[urea\right]-\left[urea\right]1/2\right)}}{1+e^{mD-N\left(\left[urea\right]-\left[urea\right]1/2\right)}}$$

### 3.4. Stopped-Flow Experiments

Single mixing folding experiments were carried out on a Pi-star stopped-flow instrument (Applied Photophysics, Leatherhead, UK). The experiments were performed at 37 °C in 50 mM Hepes buffer and 300 mM NaCl at pH 7.4; urea was used as a denaturant. Refolding and unfolding were initiated by an 11-fold dilution of the denatured or the native protein with the appropriate buffer; final protein concentrations were typically 3 µM. The excitation wavelength was 280 nm, and the fluorescence emission was measured using a 320 nm cut-off glass filter. Each experimental trace was the average of 4–6 experiments. The fluorescence time courses obtained for the unfolding, from 8 M to 4 M urea, were satisfactorily fitted by using a single exponential equation. The fluorescence time courses obtained for the unfolding from 4 M to 2 M urea, and for the refolding, were fitted by using a double exponential equation. The slow phase of the chevron plot that was obtained for the pseudo-wt C-SH2 C656S was fitted using Equation (2):

(2) $$k_{obs}=k_{f}\cdot e^{\left(-m_{f}\cdot \frac{\left[urea\right]}{RT}\right)}+k_{u}\cdot e^{\left(m_{u}\cdot \frac{\left[urea\right]}{RT}\right)}$$

The fast phase of the chevron plot that was obtained for the pseudo-wt C-SH2 C656S and the chevron plot that was obtained for the double mutant C-SH2 C656S–P684A were fitted using Equation (3):

(3) $$k_{obs}=k_{f}\cdot e^{\left(-m_{f}\cdot \frac{\left[urea\right]}{RT}\right)}+\frac{k_{u}\cdot e^{\left(m_{u1}\cdot \left[urea\right]/RT\right)}}{1+Kp\cdot e^{\left(\frac{m_{u2}}{m_{u1}}\cdot \left[urea\right]\right)}}$$

where *k_f_* and *k_u_* are the folding and unfolding rate constants, respectively; *m_f_* and *m*_*u*1_ are their associated *m* values; and *K_p_* is the partition constant between the two transition states.

Double-jump interrupted unfolding experiments were carried out on an SX18-MV stopped-flow instrument (Applied Photophysics) in 50 mM Hepes buffer at pH 7.4 and 37 °C. The excitation wavelength was 280 nm and the fluorescence emission was measured using a 320 nm cut-off glass filter. In the first step, the denatured protein (15 uM in 50 mM Hepes buffer at pH 7.4, 4 M urea) was mixed in a 1:1 dilution with refolding buffer (50 mM Hepes, pH 7.4). The result of the first mix (7.5 uM protein in 50 mM Hepes buffer at pH 7.4, 2 M urea) was mixed in a 1:1 dilution with an unfolding solution (50 mM Hepes at pH 7.4, 4 M urea). Different delay times (from 0.02 s to 180 s) were measured between the first and the second mix.

## 4. Conclusions

The effect of peptidyl prolyl cis-trans isomerization on protein folding events has been previously characterized [18,20,21,22]. In fact, whilst the cis conformer is rarely observed in the native state of proteins, it may be found frequently in their denatured states [23]. Since the isomerization of such a peptide bond might be slower than folding, the presence of proline in the primary structure typically leads to slow refolding phases. These phases present an apparent rate constant that might either be independent from the denaturant concentration or follow a dependence that may parallel the folding branch [21]. In the case of the SH2 domains, because a proline in the cis conformation is present in the native state, folding appears to be rather complex. In fact, we recently observed that the N-terminal SH2 domain from p85 presents a highly peculiar chevron, with the folding and unfolding arms showing a clear mismatch and the *k*_obs_ obtained from refolding experiments being at least 1 order of magnitude lower than those expected from unfolding experiments [27]. In this context, it is interesting to observe how the folding kinetics of C-SH2 differ from the other cases. In fact, to the best of our knowledge, this case represents the first example of the accumulation of an on-pathway intermediate, which is determined by the peptidyl prolyl cis-trans isomerization. Furthermore, this intermediate appears to be substantially populated at native conditions, as suggested earlier by NMR analysis of the domain, determining an apparent structural heterogeneity of the native state [39]. Future work based on protein engineering will further elucidate the mechanism of folding of this protein domain.

## Figures and Tables

**Table 1:** Folding kinetics parameters for C-SH2 (fast phase, λ1 and slow phase, λ2) and P684A.

Phase	*k*_f_ (s^−1^)	*m*_f_ (kcal·M^−1^·mol^−1^)	*k*_u_ (s^−1^)	*m*_u_ (kcal·M^−1^·mol^−1^)	*K*_p_ (s^−1^)	*m*_u_ (kcal·M^−1^·mol^−1^)
λ1	19.24 ± 2.15	0.81 ±0.07	0.030 ± 0.003	0.76 ± 0.33	0.020 ± 0.008	0.66 ± 0.26
λ2	4.70 ± 0.60	1.47 ± 0.27	0.0030 ± 0.0008	0.86 ± 0.1		
P684A	30.74 ± 4.12	0.98 ± 0.09	0.080 ± 0.008	0.70 ± 0.30	0.020 ± 0.003	0.64 ± 0.21
